# Expression of immune checkpoints and T cell exhaustion markers in early and advanced stages of colorectal cancer

**DOI:** 10.1007/s00262-020-02593-w

**Published:** 2020-05-11

**Authors:** Reem Saleh, Rowaida Z. Taha, Salman M. Toor, Varun Sasidharan Nair, Khaled Murshed, Mahwish Khawar, Mahmood Al-Dhaheri, Mahir Abdulla Petkar, Mohamed Abu Nada, Eyad Elkord

**Affiliations:** 1 Cancer Research Center, Qatar Biomedical Research Institute (QBRI), Hamad Bin Khalifa University (HBKU), Qatar Foundation (QF), P.O. Box: 34110, Doha, Qatar; 2 Department of Pathology, Hamad Medical Corporation, Doha, Qatar; 3 Department of Surgery, Hamad Medical Corporation, Doha, Qatar

**Keywords:** Colorectal cancer, Immune checkpoints, T cell exhaustion, Prognostic biomarker, Therapeutic target

## Abstract

Despite recent advances in colorectal cancer (CRC) treatment, a large proportion of patients show limited responses to therapies, especially in advanced stages. There is an urgent need to identify prognostic biomarkers and/or therapeutic targets in advanced stages, aiming to improve the efficacy of current treatments. We aimed to determine prognostic biomarkers in tumor tissue and circulation of CRC patients, with a special focus on T cell exhaustion markers. We found that mRNA levels of PD-1, TIM-3, CTLA-4, TIGIT, CD160, CD244, KLRG1, TOX2, TOX3, Ki-67, and PRDM1 were elevated in CRC tumor tissues. We also investigated differences in gene expression between early and advanced disease stages. We found that TOX and potentially TIM-3, CTLA-4, VISTA, TIGIT, KLRG1, TOX2, SIRT1, Ki-67, and Helios mRNA levels in tumor tissue were elevated in advanced disease stages, suggesting their potential roles in CRC progression. In contrast, PD-1 and CD160 levels in tumor tissue were downregulated in advanced stages. In the circulation of CRC patients, mRNA levels of PD-1, VISTA and LAG-3 were higher than those of healthy individuals. Moreover, in circulation, PD-1, CTLA-4 and TIGIT mRNA levels were reduced in advanced stages. Interestingly, levels of PD-1 in both tumor tissue and circulation were reduced in advanced stages, suggesting that targeting PD-1 in patients with advanced stages could be less effective. Altogether, these findings suggest some potential T cell exhaustion markers that could be utilized as prognostic biomarkers and/or therapeutic targets for CRC. However, further investigations and validations in larger cohorts are required to confirm these findings.

**Electronic supplementary material:**

The online version of this article (10.1007/s00262-020-02593-w) contains supplementary material, which is available to authorized users.

## Introduction

Cancer immunotherapies, including recombinant cytokines, immune checkpoint inhibitors (ICIs), adoptive T cell therapies, and vaccines, have revolutionized the treatment of several solid tumors and hematologic malignancies [1]. Approved ICIs in the form of monoclonal antibodies against cytotoxic T-lymphocyte–associated antigen 4 (CTLA-4), programmed cell death protein 1 (PD-1), or its ligand PD-L1 have enhanced clinical responses in patients with advanced melanoma, lung, kidney, bladder cancer; however, limited response rates were evident in many other patients due to primary or acquired resistance [2, 3]. The sensitivity of host response to ICIs is largely dependent on the tumor type and preexisting anti-tumor immunity [3]. Combining ICIs with other therapeutic agents targeting T cell exhaustion holds the potential to restore more potent anti-tumor immune responses, enhance the clinical efficacy of current treatments and overcome the resistance currently seen in many cancer patients [4].

As cancer cells divide, they acquire genetic and epigenetic alterations giving rise to new cancer clones with different molecular profiles and inheritable traits favoring tumor growth/survival and immune response escape [5]. These alterations can influence the expression of immune checkpoints (ICs), some of which might lead to T cell exhaustion [6, 7]. T cell exhaustion is a status characterized by the loss of effector function, including proliferation, release of cytokines, and secretion of cytolytic molecules, due to continuous antigen stimulation in pathological contexts, such as chronic inflammatory conditions, viral infections, and cancer [7]. Immunosuppressive cells and cytokines present in the TME can also contribute to this exhausted phenotype [6]. Additionally, T cell exhaustion is induced by the overexpression of multiple inhibitory ICs, along with other T cell exhaustion markers, such as transcription factors [8, 9]. Inhibitory ICs, such as PD-1, CTLA-4, T cell immunoglobulin and mucin domain-containing protein 3 (TIM-3), lymphocyte-activation gene 3 (LAG-3), V-domain Ig suppressor of T cell activation (VISTA), T cell immunoreceptor with Ig and ITIM domains (TIGIT), CD160, CD244, and killer cell lectin-like receptor subfamily G member 1 (KLRG1), can induce T cell dysfunction/exhaustion via direct mechanisms through the interactions with their ligands, leading to impaired T cell activation, inhibition of T cell proliferation and impaired cytokine release [8, 10–12]. Thymocyte selection-associated high-mobility-group box (TOX) family members and B lymphocyte-induced maturation protein-1 (Blimp1) are transcription factors, which can indirectly lead to immune evasion and impaired T cell activation by altering cell differentiation and gene expression [9, 13, 14]. Other markers, such as SIRT1, Ki-67 and Helios, can also influence T cell function and induce immune evasion via indirect mechanisms, and have been implicated in cancer progression [15–18]. However, little is known about the contribution of some of these markers to CRC progression and metastasis, and how their expressions differ during early and advanced stages of the disease.

In this study, we investigated gene expression levels of various inhibitory ICs including PD-1, TIM-3, LAG-3, VISTA, CTLA-4, TIGIT, CD160, CD244, and KLRG1 in the tumor tissue of CRC patients. In addition, expression levels of markers related to T cell exhaustion, cell survival, senescence, proliferation and differentiation; for example, members of the TOX family, silent information regulator 1 (SIRT1), PRDM1 (gene name for Blimp1), Helios and Ki-67, were also determined in tumor tissues of CRC patients. These genes were selected based on their importance in cancer progression and T cell exhaustion as implicated by preclinical models and clinical data; however, their expression profiles in CRC and their expression patterns during the onset and progression of disease have not been addressed yet. Gene expression analyses to determine differences between early and advanced disease stages could be a useful tool to provide insights into the transcriptomic changes during disease onset and progression [19, 20]. Overall, our data provide novel insights into some of immune signature genes, which are altered during early and advanced stages of CRC and could be utilized as prognostic biomarkers and/or therapeutic targets for CRC.

## Materials and methods

### Sample collection and storage

PBMCs were isolated from peripheral blood samples of 30 healthy donors (HD) and 68 CRC patients by density gradient centrifugation using Histopaque-1077 (Sigma-Aldrich, Missouri, USA). PBMCs were frozen in cryovials at a density of 5–10 × 10^6^ cells in 1 ml of freezing medium [(10% dimethylsulfoxide (DMSO; Sigma-Aldrich), 50% fetal calf serum (FCS; Hyclone, GE Healthcare Life Sciences, Utah, USA), and 40% RPMI-1640 medium (Life Technologies, New York, USA)], and stored in liquid nitrogen to be used in batches in subsequent experiments. Tumor tissues (TT) and paired-adjacent normal tissues (NT) were obtained from 70 CRC patients who underwent surgery at Hamad Medical Corporation, Doha, Qatar. TT and NT sections were identified by microscopic pathological examination by the pathologist. Typically, TT specimens were taken from tumor core, while normal tissue sections were taken away from the tumor margins and as deemed relevant by the pathologist. Tissue specimens were cut into pieces, snap-frozen in liquid nitrogen, and stored at − 80 °C to be used in subsequent experiments.

All patients included in the study were treatment-naïve prior to surgery and provided written informed consent prior to sample collection. Table 1 shows the details for the healthy donors included in this study and the clinical and pathological characteristics of all participating patients. All experiments were performed in accordance with relevant guidelines and regulations. This study was executed under ethical approvals from Hamad Medical Corporation, Doha, Qatar (protocol no. MRC-02-18-012) and Qatar Biomedical Research Institute, Doha, Qatar (Protocol No. 2017-006 and 2018-018).

**Table 1:** Characteristic features of study populations

	Healthy donors (HD)	CRC patients (tissue samples)	CRC patients (PBMC samples)
Number	30	70	68
Age (median)	30 (20–47)^§^	59 (23–96)^§^	60 (23–96)^§^
Gender (Male: Female)	14:16	47:23	42:26
TNM stage			
I		5	10
II		24	23
III		31	24
IV		10	11
Tumor budding			
Low		31	29
Intermediate		20	19
High		19	20

*CRC* colorectal cancer

^§^Median range

### RNA extraction and reverse transcription

Total RNA was extracted from PBMC of HDs and CRC patients, and NT/TT of CRC patients, using RNA/DNA/Protein Purification Plus Kit (Norgen, Ontario, Canada). Briefly, PBMCs were washed with 1 × phosphate-buffered saline (1 × PBS) and resuspended with lysis buffer. TTs and NTs were also resuspended with lysis buffer, but homogenized using a tissue homogenization set (Bioneer Inc., Daejeon, South Korea). RNA extraction was performed following the manufacturer’s protocol. RNA concentrations were measured using NanoDrop 2000 (Thermo scientific). 1 µg of RNA was reverse-transcribed into cDNA using QuantiTect Reverse Transcription Kit (Qiagen, Hilden, Germany).

### Quantitative real-time reverse transcriptase PCR (qRT-PCR)

qRT-PCR was performed using QuantStudio 6/7 Flex real-time PCR system (Applied Biosystems, California, USA), for the genes listed in Supplementary Table 1, with PowerUp SYBR Green Master Mix (Applied Biosystems). Quantification of relative gene expression was determined using 2^−ΔΔCT^, and normalized to β-actin. Sequences for the primers are shown in Supplementary Table 1.

For circulation, the *C*_t_ values of target gene from the HDs were initially normalized to β-actin. The relative expression of target gene from each patient was calculated by normalizing the expression of each sample with average expression of the same gene from HDs, and subsequently 2^−ΔΔCT^ method was applied. Additionally, for CRC tissues, the relative expression was calculated by normalizing the expression of each TT sample to its corresponding NT after the initial normalization to β-actin. Therefore, the relative fold change of each TT sample was calculated by keeping the value of corresponding NT sample as constant “1”. The statistical significance was calculated by performing unpaired and paired *t* tests for circulation and CRC tissues, respectively [21, 22].

### Statistical analyses

Statistical analyses were performed using GraphPad Prism 8 software (GraphPad Software, California, USA). Paired and unpaired *t* tests were performed on samples that passed the Shapiro–Wilk normality test. Non-parametric Wilcoxon signed-rank tests or Mann–Whitney *U* tests were performed for samples that did not show normal distribution. Mann–Whitney *U* test was used to compare the differences between early (stages I and II) and advanced (stages III and IV) stages. Comparisons of gene expression among different tumor budding grades were performed using Kruskal–Wallis test one-way analysis of variance (ANOVA). A *P* value of > 0.05 was considered statistically nonsignificant. The *P* values are represented as follows;***P < 0.001,**P < 0.01,*P ≤ 0.05. Data are presented as mean ± standard error of the mean (SEM).

## Results

### Elevated expression of immune checkpoints and T cell exhaustion-related genes in the tumor tissue of CRC patients

**Fig. 1:**
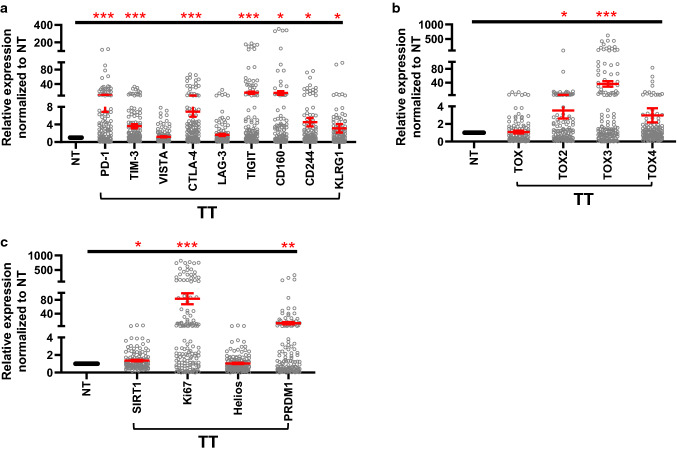
Gene expression of immune checkpoints, T cell exhaustion-related markers and cancer progression-related markers in colorectal cancer tissues. RNA was isolated from normal and tumor tissues of 70 patients, and reverse transcribed to cDNA. Quantitative RT-PCR was performed to assess the expression levels of immune checkpoints (PD-1, CTLA-4, TIM-3, VISTA, CTLA-4, LAG-3, TIGIT, CD160, CD244, and KLRG1) (**a**), TOX family members (TOX, TOX2, TOX3, and TOX4) (**b**), and T cell exhaustion-related markers (SIRT1, Ki-67, Helios and PRDM1) (**c**). Scatter plots show expression levels in TT normalized to NT. The relative expression of each gene was calculated by normalization with β-actin. Each dot represents the relative expression value of a particular gene of an individual patient. Mean ± standard error of the mean (SEM) is shown in red

Increased expression of multiple immune checkpoints and T cell exhaustion markers in the tumor tissue of cancer patients has been associated with the suppression of anti-tumor immunity and resistance to therapy [3, 23]. Therefore, we investigated the expression levels of different genes that have been previously implicated in immune evasion and T cell exhaustion in the tumor tissue (TT) and paired-normal tissue (NT) of CRC patients (Fig. 1). The panel of genes consisted of well-known inhibitory ICs, PD-1, TIM-3, CTLA-4, VISTA, LAG-3, TIGIT, CD244, CD160, and KLRG1 [24], in addition to T cell exhaustion-related markers namely TOX, TOX2, TOX3, TOX4 [9, 14], Helios, PRDM1 (Blimp1), Ki-67 [25], and other markers involved in tumorigenesis such as SIRT1 [15].

We found that mRNA levels of PD-1, TIM-3, CTLA-4, TIGIT, CD160, CD244, and KLRG1 were significantly higher in TT, compared to NT (Fig. 1a). LAG-3 and VISTA mRNA levels were relatively similar in TT and NT (Fig. 1a). We also investigated the expression of TOX family members and other tumorigenic markers. We found that TOX mRNA level was relatively similar in TT and NT, while TOX2 and TOX3 mRNA levels were significantly higher in TT (Fig. 1b). TOX4 mRNA level was upregulated in TT compared with NT; however, it did not reach statistical significance (Fig. 1b). SIRT1, Ki-67, and PRDM1 mRNA levels were also upregulated in TT (Fig. 1c); however, mRNA levels of Helios in TT and NT were relatively similar (Fig. 1c).

### Comparative analyses of ICs and T cell exhaustion markers in tumor tissues from CRC patients with early and advanced stages

**Fig. 2:**
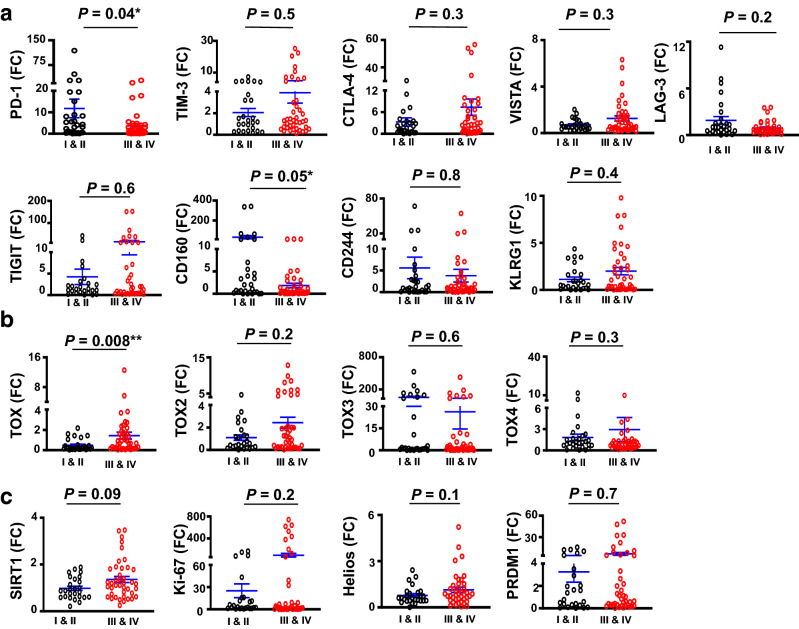
Gene expression of immune checkpoints, and T cell exhaustion and cancer progression-related markers in colorectal cancer tissues from patients at early and advanced disease stages. Scatter plots show the expression of markers in early (I & II) and advanced (III & IV) disease stages (CRC patients *n* = 70, stage I, *n* = 5; stage II, *n* = 24; stage III, *n* = 30; stage IV, *n* = 11). Scatter plots show the expression levels of immune checkpoints (PD-1, TIM-3, CTLA-4, VISTA, LAG-3, TIGIT, CD160, CD244 and KLRG1) (**a**); TOX family members (TOX, TOX2, TOX3 and TOX4) (**b**); other T cell exhaustion markers (SIRT1, Ki-67, Helios and PRDM1) (**c**) in tumor tissues of CRC patients. Results are presented as fold change (FC) of gene expression in TT versus NT. Means ± standard error of the means (SEM) are depicted on the scatter plots

Tumor tissues are heterogeneous; different tumor types and at various pathological stages can express different levels of inhibitory ICs, transcription factors and other molecules to escape immunosurveillance and promote tumor growth [26]. Therefore, we investigated whether there were any differences in mRNA levels of different genes between early and advanced disease stages (Fig. 2). We found that PD-1 mRNA level was significantly higher in early stages, compared to advanced stages (11.8 ± 4.5 vs. 3.6 ± 6.5, Fig. 2a). Although TIM-3, CTLA-4, VISTA, and TIGIT mRNA levels were increased in advanced stages, results did not reach statistical significance (Fig. 2a). LAG-3 mRNA level showed a trend towards increased expression in early disease stages, compared to advanced stages (Fig. 2a). Similarly, CD160 mRNA level was increased in early stages, compared to advanced stages (30.9 ± 16.2 vs. 1.8 ± 0.5, Fig. 2a). CD244 did not show any differences with disease stages, while KLRG1 showed a trend towards increased expression in advanced stages (Fig. 2a). Notably, TOX mRNA level was significantly increased in advanced stages, compared to early stages (1.4 ± 0.33 vs. 0.49 ± 0.11, Fig. 2b). TOX2 showed a trend towards increased expression in advanced stages (Fig. 2b), while TOX3 and TOX4 mRNA levels did not show any differences within disease stages (Fig. 2b). Although mRNA levels of SIRT1, Ki-67, Helios, and PRDM1 were increased in advanced stages, they did not show statistical significance (Fig. 2c).

Together, these data suggest that TOX, and potentially TIM-3, CTLA-4, VISTA, TIGIT, KLRG1, TOX2, SIRT1, Ki-67, and Helios mRNA levels in CRC tumor tissues increase in advanced disease stages (Table 2), suggesting their possible roles in CRC progression. On the other hand, PD-1 and CD160 mRNA levels were higher in CRC tumor tissues from patients with early stages, indicating that targeting these two ICs in early stages could be more effective than in advanced stages.

**Table 2:** Summary for gene expression and comparative analyses in the tumor tissue and circulation of CRC patients with early and advanced stages

Gene	Level in CRC TT compared to NT	Gene level in advanced stages compared to early stages (TT)	Level in CRC PBMC compared to healthy donors	Gene level in advanced stages compared to early stages (circulation)
PD-1	Upregulated***	Downregulated*	Upregulated***	Downregulated*
TIM-3	Upregulated***	Upregulated (n.s)	No change	No change
VISTA	No change	Upregulated (n.s)	Upregulated***	No change
CTLA-4	Upregulated***	Upregulated (n.s)	Upregulated (n.s)	Downregulated*
LAG-3	No change	Downregulated (n.s)	Upregulated***	No change
TIGIT	Upregulated***	Upregulated (n.s)	Downregulated**	Downregulated**
TOX	No change	Upregulated**	Downregulated***	No change
TOX2	Upregulated*	Upregulated (n.s)	Upregulated (n.s)	No change
TOX3	Upregulated***	No change	N.A	N.A
TOX4	Upregulated (N.S)	No change	N.A	N.A
SIRT1	Upregulated*	Upregulated (n.s)	Downregulated***	No change
Ki-67	Upregulated***	Upregulated (n.s)	N.A	N.A
Helios	No change	Upregulated (n.s)	N.A	N.A
PRDM1	Upregulated**	Upregulated (n.s)	N.A	N.A
KLRG1	Upregulated*	Upregulated (n.s)	N.A	N.A
CD160	Upregulated*	Downregulated*	N.A	N.A
CD244	Upregulated*	No change	N.A	N.A

*CRC* colorectal cancer, *TT* tumor tissue, *PBMC* peripheral blood mononuclear cells, *N.S* not significant, *N.A* not available

***P < 0.001, **P < 0.01, *P ≤ 0.05

### Expression of immune checkpoints and T cell exhaustion-related genes in the circulation of CRC patients

Increased expression of immune checkpoints and T cell exhaustion markers in the circulation of colorectal cancer patients have been associated with the suppression of anti-tumor immunity [27]. Due to the limited RNA obtained from patient samples, we had to prioritize the genes and select a subset of genes from the panel of genes included in CRC tissue investigations, and determine their expression levels in the peripheral blood mononuclear cells (PBMCs) of CRC patients. We selected the most common ICs, some of which are being targeted in ongoing clinical trials or are approved for cancer immunotherapy, including TIM-3, LAG-3, TIGIT, VISTA, CTLA-4 and PD-1 [2]. In addition, we selected some markers that could be of interest, given their recent emerging roles in T cell exhaustion, such as TOX and TOX2 [14], and SIRT1, which has been implicated in CRC progression [15, 28].

**Fig. 3:**
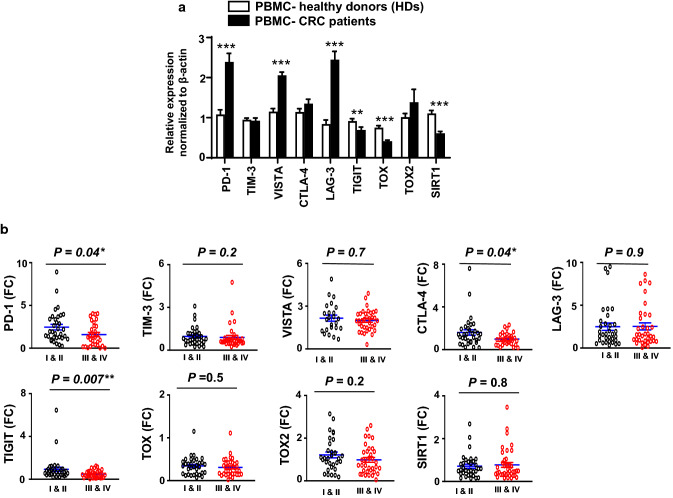
Gene expression of immune checkpoints, T cell exhaustion and cancer progression-related markers in the circulation of colorectal cancer patients at early and advanced disease stages. RNA was isolated from peripheral blood mononuclear cells (PBMCs) of 30 healthy donors and 68 CRC patients, and reverse transcribed to cDNA. Quantitative RT-PCR was performed to assess the expression levels of immune checkpoints (PD-1, TIM-3, VISTA, CTLA-4, LAG-3, and TIGIT) and T cell exhaustion markers (TOX, TOX2 and SIRT1). Bar plot shows relative expression of each gene, normalized to average expression level recorded for healthy donors (**a**). Scatter plots show the expression levels of PD-1, TIM-3, VISTA, CTLA-4, LAG-3, TIGIT, TOX, TOX2, and SIRT1 between early (I & II) and advanced (III & IV) disease stages (stage I, *n* = 10; stage II, *n* = 23; stage, III *n* = 24; stage IV, *n* = 11) (**b**). Results are presented as fold change (FC) of gene expression in CRC versus HD. Results are presented as mean ± standard error of the mean (SEM)

We found that the mRNA expression levels of PD-1, VISTA, and LAG-3 were significantly elevated in the circulation of CRC patients, compared to healthy individuals (Fig. 3a). A trend towards an upregulated level of TOX2 was seen in the circulation of CRC patients. TIM-3 mRNA levels were relatively similar in both groups (Fig. 3a). Although CTLA-4 mRNA level was elevated in the circulation of CRC patients, it did not reach statistical significance (Fig. 3a). In contrast, TIGIT, TOX and SIRT1 mRNA levels were significantly reduced in the circulation of CRC patients (Fig. 3a). Next, we investigated differences in the expression levels of these genes between early and advanced disease stages in the circulation of CRC patients. PD-1, CTLA-4, and TIGIT expression levels were significantly higher in early stages compared with advanced stages (2.5 ± 0.3 vs. 1.6 ± 0.3, 1.6 ± 0.3 vs. 0.9 ± 0.1, and 0.9 ± 0.02 vs. 0.4 ± 0.1, respectively, Fig. 3b). Levels of other genes including TIM-3, VISTA, TOX, TOX2 and SIRT1 were relatively similar in early and advanced stages (Fig. 3b), possibly suggesting that their expressions in the circulation have no prognostic value.

### Increased expression of TIM-3, CTLA-4, TIGIT, TOX, and SIRT1 mRNA in the tumor tissue compared to the circulation of CRC patients

**Fig. 4:**
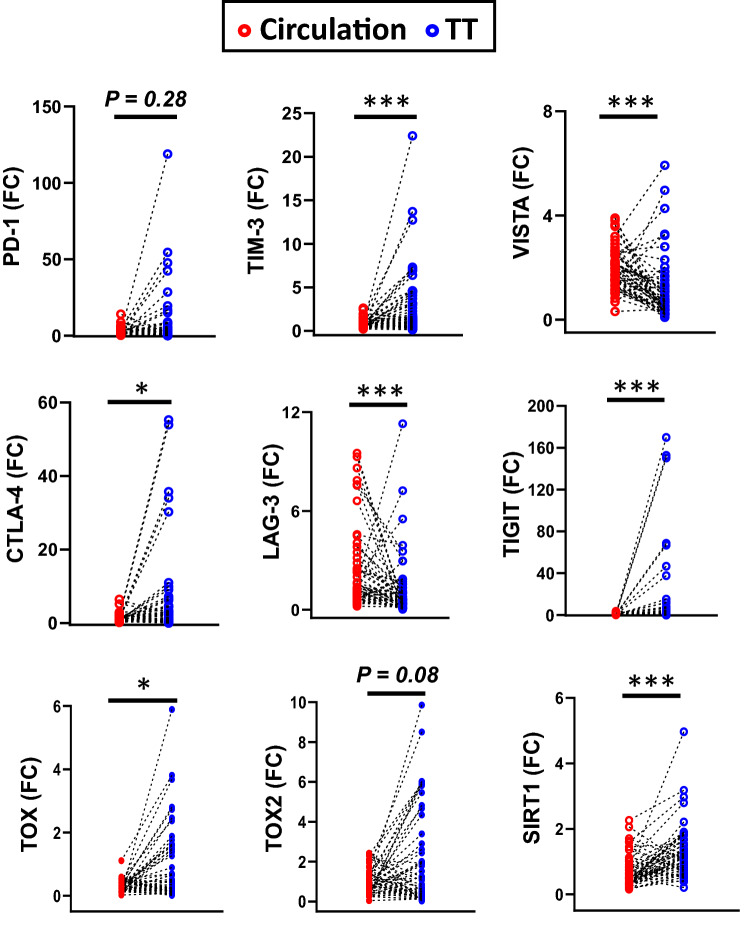
Paired comparison of gene expression levels in the circulation and tumor tissues of CRC patients. Scatter plots show the paired comparison between the circulation and TT of 56 CRC patients based on the expression level of PD-1, TIM-3, VISTA, CTLA-4, LAG-3, TIGIT, TOX, TOX2, and SIRT1. Results are presented as fold change (FC) of gene expression in TT versus NT for tissue and CRC versus HD for circulation

Since we detected the levels of common T cell exhaustion markers in the tumor tissue and circulation of CRC patients, we performed a paired comparison between the circulation and tumor tissues based on the level of these genes (Fig. 4). Genes including TIM-3, CTLA-4, TIGIT, TOX, and SIRT1 showed higher levels in the TME, compared with circulation. PD-1 and TOX2 showed a trend towards an upregulated level in the TME, compared to circulation. Only two genes (VISTA and LAG-3) showed different patterns, where levels were significantly higher in the circulation, compared with the TME.

Taken together, these data indicate the differential expression of T cell exhaustion markers in the TME and circulation of CRC patients (summarized in Table 2). This may suggest that elevated expression of PD-1, TIM-3, CTLA-4, TIGIT, TOX, TOX2, and SIRT1 genes in the TME of CRC is induced by tumor-associated, factors, unlike VISTA, and LAG-3.

### Comparative analyses of marker expression in the tumor tissue of CRC patients with different grades of tumor budding

Tumor budding, evidenced by the presence of individual cells and small clusters of tumor cells at the invasive front of carcinomas, has gained attention, particularly in CRC [29]. Tumor budding is an independent prognostic marker for CRC and important indicator of increased risk of nodal metastasis and poor disease outcomes [30]. Some clinicians and pathologists are considering that the use of tumor budding could be more helpful than using TNM staging in patient stratification into risk categories [29]. TNM staging and tumor budding represent different clinicopathological measures; they are independent of each other with different prognostic values.

Here, we investigated whether there are any differences in mRNA levels of genes investigated in TT with the grade of tumor budding, classified as low/intermediate/high (Supplementary Figure 1). PD-1, TIM-3, CTLA-4, LAG-3, and CD244 mRNA levels were relatively similar amongst the different grades of tumor budding (Supplementary Figure 1A). VISTA, TIGIT and KLRG1 showed a trend towards increased expression in patients with high grades of tumor budding (Supplementary Figure 1A). However, level of CD160 mRNA was significantly increased in patients with low grades of tumor budding (Supplementary Figure 1A). TOX, TOX2, TOX3, and TOX4 (Supplementary Figure 1B), in addition to SIRT1, Ki-67, Helios, and PRDM1 mRNA levels (Supplementary Figure 1C) showed no differences with grades of tumor budding.

Collectively, these data suggest that mRNA levels of VISTA, TIGIT and KLRG1 in CRC tumor tissues could be elevated in CRC patients with high grades of tumor budding, which is considered as a risk factor for poor disease prognosis and metastasis in CRC patients. On the other hand, CD160 mRNA level was increased in patients with low grades of tumor budding. These data indicate that some IC expressions could be associated with tumor metastasis.

## Discussion

Increased expression of T cell exhaustion markers, including ICs, in many cancer types has been previously reported [8]. We have previously reported that mRNA levels of some ICs, including PD-1, TIM-3, CTLA-4, and TIGIT, were elevated in the circulation and TME of CRC patients [21, 31]. In this study, we extended this investigation by including other T cell exhaustion markers, and determined their transcriptomic expressions in the TME and circulation of CRC patients at early and advanced disease stages to identify potential prognostic biomarkers and/or therapeutic targets for CRC patients. Transcriptomic changes of these markers with pathological stages and tumor budding grades in CRC patients have not been studied before.

We found that mRNA levels of PD-1, TIM-3, CTLA-4, TIGIT, CD160, CD244, and KLRG1 were elevated in CRC tumor tissue, implicating their potential contribution to CRC development and/or progression. Signaling pathways of these ICs have been associated with increased suppressive activity of T regulatory cells (Tregs), impaired activation of antigen-presenting cells and T effector cells, inhibition of T cell proliferation and reduced cytokine production [2, 3]. In addition, the importance of PD-1 and CTLA-4 [32–34], TIM-3 [35], CD160 [12], and CD244 [11] in promoting tumor growth and progression has been reported in preclinical models. Zhou et al*.,* showed that targeting TIGIT, using CRISPR/Cas9 system, in mouse colon cancer models inhibited tumor growth [36]. Greenberg et al*.,* showed that KLRG1 positively regulates tumor growth/metastasis in animal models of breast and colon cancers, and melanoma, while its blockade enhances anti-tumor immunity [37].

We also found that mRNA levels of TOX2 and TOX3 were significantly higher in CRC tumor tissues, suggesting their potential roles in CRC development and/or progression. The role of TOX proteins in T cell exhaustion has recently been highlighted in several studies [9, 13, 14, 38]. Additionally, there are no studies on the expression of TOX2 and TOX3 in CRC tumor tissues. It was demonstrated that TOX and TOX2 can cooperatively work together to induce T cell exhaustion by upregulating IC expression in chimeric antigen receptor (CAR) T cell model, and their targeting was shown to be beneficial in enhancing anti-tumor immune responses and reducing tumor growth [14].

SIRT1, Ki-67, and PRDM1 mRNA levels were also elevated in CRC tumor tissues; these markers could contribute to CRC by promoting tumorigenesis, and inducing T cell exhaustion and functional impairment via indirect mechanisms [15–17, 39]. SIRT1 is a member of the sirtuins family; acts as a negative regulator for apoptosis, IL-2 production, anti-tumor T cell responses and the tumor suppressor gene p53 [40], and positively regulates tumor growth [15, 28]. High Ki-67 index has been associated with CD8^+^ T cell exhausted phenotype in tumors [17], suggesting that increased proliferative activity is associated with functional impairment of T cells [16]. Moreover, silencing Ki-67 gene in vitro and in vivo has been shown to induce tumor cell apoptosis [41]. Blimp-1, encoded by PRDM1 gene, has also been implicated in T cell exhaustion and associated with the induction of inhibitory IC expression (such as PD-1 and TIGIT), IL-10 production and effector T cell death [39]. In addition, overexpression of Blimp-1 in breast cancer models has been associated with tumor invasiveness and metastasis [42]. The expression of Helios in CRC tumor tissues has been linked with increased stability and suppressive activity of Tregs, which in turn positively regulate cancer progression [18]. Furthermore, the expression of Helios has been shown in exhausted CD4^+^ T cells [43]; however, the exact pathway(s) by which Helios induces T cell exhaustion is yet to be determined.

Additionally, we found that PD-1 mRNA levels in CRC tumor tissue and circulation were higher in early stages, suggesting that targeting PD-1 in CRC patients with advanced stages could be less effective. In contrast to the TME, the mRNA levels of CTLA-4 and TIGIT in the circulation of CRC patients at early stages were higher than those at advanced stages. This could suggest that T cells expressing CTLA-4 and TIGIT accumulate in the circulation of CRC patients during the onset of disease, until they are recruited to the TME as disease progresses, resulting in their accumulation within the tumor tissue.

Levels of TIM-3, CTLA-4, TIGIT, TOX, TOX2, and SIRT1 genes in tumor tissue were significantly higher than that of the circulation; their expressions within the TME could be induced by tumor-mediated immunosuppression [3, 26]. These data implicate the potential importance of their elevated expression in the TME and their potential contribution to cancer progression. In support of this, the association of CRC progression with TIM-3 [44, 45] and SIRT1 [15, 28] expression in tumor tissues have been reported.

To our best knowledge, our study is the first one showing the differences in the expression of some T cell exhaustion markers between early and advanced disease stages in the TME and circulation of CRC patients. Collectively, our results could offer a therapeutic benefit and help in designing more effective treatment regimens for patients with advanced stages of CRC. However, further investigations are required to validate these findings in larger cohorts of patients. Additional studies are required to elucidate the mechanisms which regulate the expression of some of these markers in the tumor tissue and circulation of CRC patients.

### Electronic supplementary material

Below is the link to the electronic supplementary material.

Supplementary Figure 1. Gene expression of immune checkpoints, T cell exhaustion and cancer progression-related markers in CRC tissues with different grades of tumor budding. Scatter plots show the expression levels of immune checkpoints (PD-1, TIM-3, CTLA-4, VISTA, LAG-3, TIGIT, CD160, CD244 and KLRG1) (**A**); TOX family members (TOX, TOX2, TOX3 and TOX4) (**B**) and other T cell exhaustion markers (SIRT1, Ki-67, Helios and PRDM1) (**C**) in CRC tissues with low (denoted as L), intermediate (denoted as I) and high (denoted as H) grades of tumor budding (CRC patients n= 70, low grade of tumor budding, n = 31; intermediate, n = 20; high, n = 19). Results are presented as fold change (FC) of gene expression in TT vs. NT. Means ± standard error of the means (SEM) are depicted on the scatter plots. (PDF 616 kb)

Supplementary file2 (PDF 90 kb)

## Abbreviations

Blimp1: B lymphocyte-induced maturation protein-1
CRC: Colorectal cancer
CTLA-4: Cytotoxic T-lymphocyte-associated protein 4
KLRG1: Killer cell lectin-like receptor subfamily G member 1
LAG-3: Lymphocyte-activation gene 3
PBMC: Peripheral blood mononuclear cell
PD-1: Programmed cell death-1
PRDM1: PR domain zinc finger protein 1
SIRT1: Silent information regulator 1
TIGIT: T cell immunoreceptor with Ig and ITIM domains
TIM-3: T-cell immunoglobulin and mucin domain-3
TME: Tumor microenvironment
TOX: Thymocyte selection-associated high-mobility group box
Treg: T regulatory cell
VISTA: V-domain Ig-containing Suppressor of T cell Activation
