# Supplementary figures and images for: Expression of immune checkpoints and T cell exhaustion markers in early and advanced stages of colorectal cancer
Source: Cancer Immunol Immunother. 2020 May 11;69(10):1989–99. doi: 10.1007/s00262-020-02593-w (PMC7511277; doi:10.1007/s00262-020-02593-w)

# Supplementary Figure 1

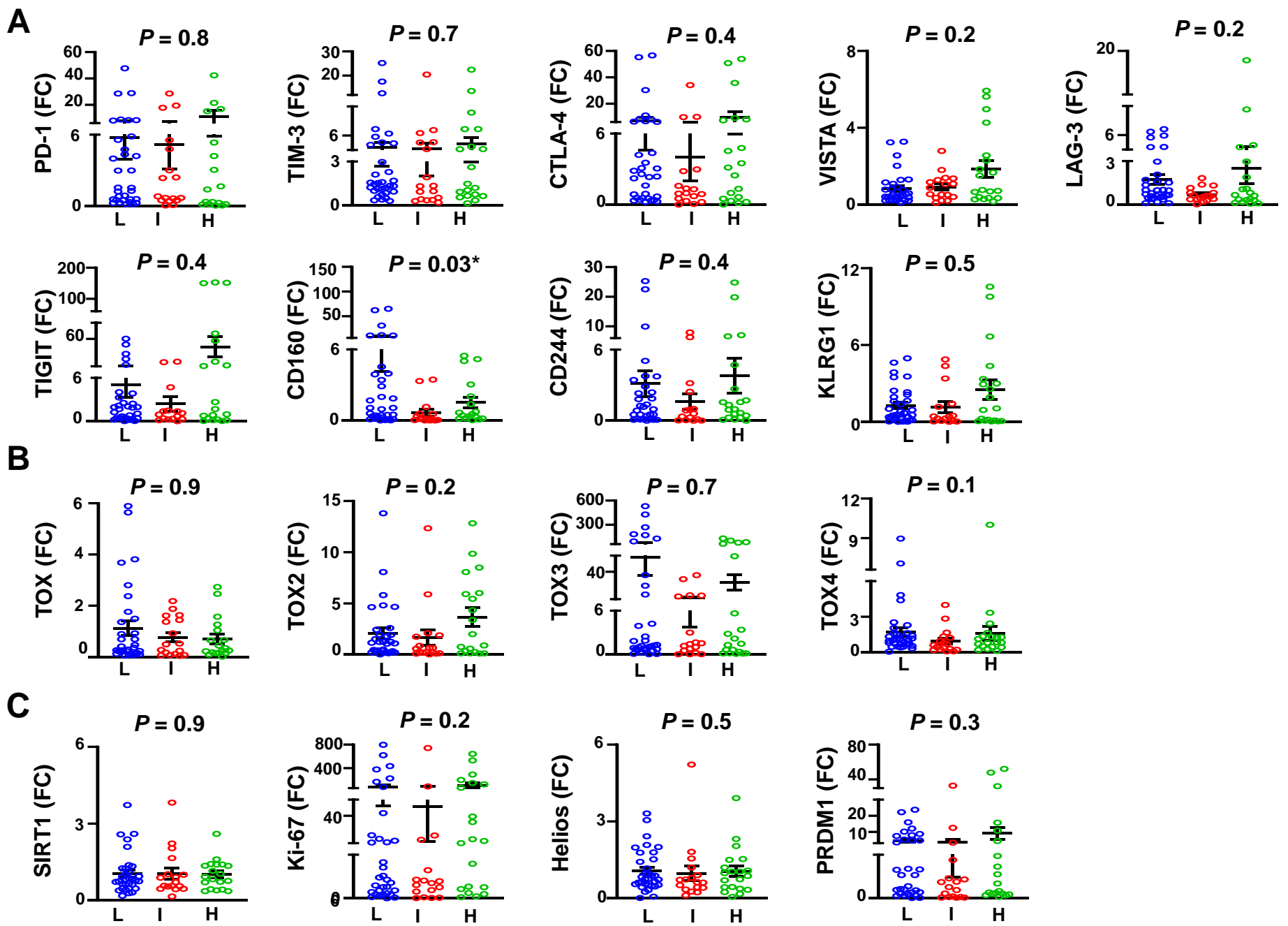

Supplement: Supplementary file 1 — Gene expression of immune checkpoints, T cell exhaustion and cancer progression-related markers in CRC tissues with different grades of tumor budding. Scatter plots show the expression levels of immune checkpoints (PD-1, TIM-3, CTLA-4, VISTA, LAG-3, TIGIT, CD160, CD244 and KLRG1) (A); TOX family members (TOX, TOX2, TOX3 and TOX4) (B) and other T cell exhaustion markers (SIRT1, Ki-67, Helios and PRDM1) (C) in CRC tissues with low (denoted as L), intermediate (denoted as I) and high (denoted as H) grades of tumor budding (CRC patients n= 70, low grade of tumor budding, n = 31; intermediate, n = 20; high, n = 19). Results are presented as fold change (FC) of gene expression in TT vs. NT. Means ± standard error of the means (SEM) are depicted on the scatter plots. (PDF 616 kb) [file 262_2020_2593_MOESM1_ESM.pdf]
