# Supplementary material for: Expression of immune checkpoints and T cell exhaustion markers in early and advanced stages of colorectal cancer
Source: Cancer Immunol Immunother. 2020 May 11;69(10):1989–99. doi: 10.1007/s00262-020-02593-w (PMC7511277; doi:10.1007/s00262-020-02593-w)
Supplement: Supplementary file 2 — Supplementary file2 (PDF 90 kb) [file 262_2020_2593_MOESM2_ESM.pdf]

## Supplementary Table 1: Primer sequences used in the study

### RT-qPCR primers

| Genes | Forward primer (5′-3′) | Reverse primer (5′-3′) |
|---|---|---|
| PD-1 | CCAGGATGGTTCTTAGACTCCC | TTTAGCACGAAGCTCTCCGAT |
| TIM-3 | TCCAAGGATGCTTACCACCAG | GCCAATGTGGATATTTGTGTTAGATT |
| CTLA-4 | GCCCTGCACTCTCCTGTTTTT | GGTTGCCGCACAGACTTCA |
| VISTA | ACGCCGTATTCCCTGTATGTC | TTGTAGAAGGTCACATCGTGC |
| LAG-3 | GCGGGGACTTCTCGCTATG | GGCTCTGAGAGATCCTGGGG |
| TIGIT | TCTGCATCTATCACACCTACCC | CCACCACGATGACTGCTGT |
| TOX | TATGAGCATGACAGAGCCGAG | GGAAGGAGGAGTAATTGGTGGA |
| TOX2 | AGAGCGAGAACAACGAAGACT | TGGCCTGATAGGAGTAGGCAG |
| TOX3 | CCTGCCAGCCTGGACTTC | GAGGAGGCGTGATTGGTGG |
| TOX4 | TGACAATTACCTGACGATCACAG | TCCAAGGAGATAGGTGGGATTTC |
| SIRT1 | TAGCCTTGTCAGATAAGGAAGGA | ACAGCTTCACAGTCAACTTTGT |
| Ki67 | ACGCCTGGTTACTATCAAAAGG | CAGACCCATTTACTTGTGTTGGA |
| Helios | TCACCCGAAAGGGAGCACT | CATGGCCCCTGATCTCATCTT |
| CD244 | TCGTGATTCTAAGCGCACTGT | CAGGTTCTTGTGACGTGGGAG |
| CD160 | GCTGAGGGGTTTGTAGTGTTT | GTGTGACTTGGCTTATGGTGA |
| KLRG1 | TCCATGTTAGAGTTGCCTACGG | AAGTGGAGTAGTTGGAGCCCT |
| PRDM1 | AAGCAACTGGATGCGCTATGT | GGGATGGGCTTAATGGTGTAGAA |
| β-actin | AGAGCTACGAGCTGCCTGAC | AGCACTGTGTTGGCGTACAG |
